# Sulforaphane Protects against Cardiovascular Disease via Nrf2 Activation

**DOI:** 10.1155/2015/407580

**Published:** 2015-10-25

**Authors:** Yang Bai, Xiaolu Wang, Song Zhao, Chunye Ma, Jiuwei Cui, Yang Zheng

**Affiliations:** ^1^The Cardiac Surgery Department, The First Hospital of Jilin University, Changchun 130021, China; ^2^The Jilin Province People's Hospital, Changchun 130021, China; ^3^The Spine Surgery Department, The First Hospital of Jilin University, Changchun 130021, China; ^4^Cancer Center, The First Hospital of Jilin University, Changchun 130021, China

## Abstract

Cardiovascular disease (CVD) causes an unparalleled proportion of the global burden of disease and will remain the main cause of mortality for the near future. Oxidative stress plays a major role in the pathophysiology of cardiac disorders. Several studies have highlighted the cardinal role played by the overproduction of reactive oxygen or nitrogen species in the pathogenesis of ischemic myocardial damage and consequent cardiac dysfunction. Isothiocyanates (ITC) are sulfur-containing compounds that are broadly distributed among cruciferous vegetables. Sulforaphane (SFN) is an ITC shown to possess anticancer activities by both *in vivo* and epidemiological studies. Recent data have indicated that the beneficial effects of SFN in CVD are due to its antioxidant and anti-inflammatory properties. SFN activates NF-E2-related factor 2 (Nrf2), a basic leucine zipper transcription factor that serves as a defense mechanism against oxidative stress and electrophilic toxicants by inducing more than a hundred cytoprotective proteins, including antioxidants and phase II detoxifying enzymes. This review will summarize the evidence from clinical studies and animal experiments relating to the potential mechanisms by which SFN modulates Nrf2 activation and protects against CVD.

## 1. Introduction

Cardiovascular disease (CVD) is a class of diseases that involve the heart or blood vessels, such as ischemic heart disease, hypertensive heart disease, and peripheral artery disease. Generally, CVD is related to hypertension, atherosclerosis, obesity, or diabetes [1]. CVD underlies an unparalleled proportion of the global disease burden and it will remain the main cause of mortality for the near future [2]. Although our understanding of the pathobiology and treatment of atherosclerosis and CVD has advanced considerably over the past 100 years and therapies continue to evolve, the global problem we face in alleviating the enormous burden of CVD is complex and multidimensional [3]. CVD is now increasingly prevalent in developing countries, as the increased affluence of industrialized societies leads to abundant calorie-dense food, access to labor-saving devices that reduce physical activity, and ready availability of tobacco products. Traditional cultures based on consumption of grains, fruits, and vegetables along with heavy physical activity and a less stressful life are being replaced by more intense urban cultures [4]. CVD is also the biggest killer in the western world, despite significant advances in the understanding of the relevant molecular mechanisms [5]. It is in the public interest to identify effective approaches to reducing the impact of CVD in both developed and developing regions [6]. Oxidative stress plays a major role in the pathophysiology of cardiac disorders [7]. High levels of oxidative stress resulting from increased cardiac generation of reactive oxygen species (ROS) are thought to contribute to contractile and endothelial dysfunction, myocyte apoptosis and necrosis, and extracellular matrix remodeling in the heart [8]. There are two types of small molecular antioxidant involved in cellular protection against oxidative and electrophile toxicities; direct antioxidants are redox-active and short-lived, necessitating replenishment or regeneration, and may also evoke prooxidant effects, while indirect antioxidants may or may not be redox-active, are not consumed, have long half-lives, and are unlikely to evoke prooxidant effects [9]. Therefore, current research focuses on the characterization and validation of health-promoting functional foods that specifically target endogenous antioxidant defenses. Epidemiological studies have indicated that cruciferous vegetable intake correlated with a decreased risk for CVD. Because broccoli is a cruciferous vegetable and contains isothiocyanates (ITC), which can produce the redox-regulated cardioprotective protein, thioredoxin (Trx), it was reasoned that consumption of broccoli could be beneficial to the heart [10]. Sulforaphane (SFN) is by far the most widely studied and best characterized ITC [11].

## 2. Structural Features and Biological Activity of SFN

**Figure 1:**
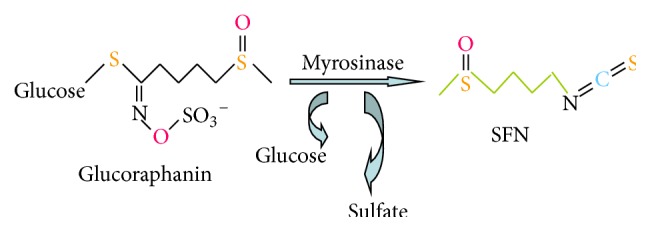
Glucoraphanin is the major glucosinolate in broccoli. Under neutral conditions, Grn is hydrolyzed by myrosinase to yield glucose, sulfate, and sulforaphane (SFN).

SFN [1-isothiocyanate-(4R)-(methylsulfinyl) butane] [12] is the hydrolysis product of glucoraphanin (Grn), the main glucosinolate (GLS) in broccoli [13, 14]. GLS (*β*-thioglucose N-hydroxysulfates) are relatively stable biosynthetic precursors along with SFN [15]. GLS exist in association with but structurally segregated from the myrosinase enzyme (*β*-thioglucoside glucohydrolase); this is released upon plant cell injury (e.g., by chewing) and cleaves GLS to produce SFN, sulfate, and glucose [16, 17] (Figure 1). Ahn et al. [18] showed that 24 synthetic sulfoxythiocarbamate analogs that retained the important structural features of SFN analogs, demonstrating efficacy for phase II enzyme induction in a range of cell lines. In order to eliminate chemicals, drug-metabolizing enzymes initially metabolize the compounds (phase I reaction) and then detoxify electrophiles and oxidants (phase II reaction). A group of heterogeneous enzymes carry out the phase II reactions including glutathione-S-transferase (GST), epoxide hydrolase, UDP-glucuronosyltransferase and sulfotransferase, and NAD(P)H: quinone oxidoreductase 1 (NQO1). The antioxidant systems of phase II reactions can detoxify ROS directly [19, 20]. GST catalyzes the conjugation of glutathione (GSH) with electrophilic compounds making them more water soluble and facilitating their removal from the body. It is well known that ITC-GSH conjugates are exported out to extracellular. This continous conjugation and efflux of the conjugate can cause intracellular GSH level to drop. ITC can also induce GST scavenge ROS [21]. Shibata et al. examined the effect of sulfhydryl molecules on cellular responses induced by 6-methylsulfinylhexyl isothiocyanate (6-HITC), an analog of SFN isolated from broccoli. Significant induction of heme oxygenase-1 (HO-1) was observed when cells were treated with 6-HITC, even in the presence of GSH [22]. It was also demonstrated that the main mechanism of action of SFN involves regulation of the nuclear factor erythroid-derived 2- (NF-E2) related factor 2- (Nrf2-) antioxidant response element (ARE) pathway; this in turn upregulates the expression of a range of antioxidant enzymes including HO-1, NQO1, GST, *γ*-glutamyl cysteine ligase (GCL), and glutathione reductase (GR) [23]. Therefore, SFN has been considered to act as an indirect antioxidant and a highly potent inducer of phase II cytoprotective enzymes. The resultant detoxification of electrophiles and oxidants can protect against carcinogens, oxidative stress, and inflammation.

## 3. Nrf2 Signaling Pathway and Antioxidant Mechanism

### 3.1. Nrf2-Kelch-Like Chicken Erythroid-Derived Cap “n” Collar Homology (ECH) Factor-Associated Protein 1- (Keap1) ARE Signaling Pathway

Nrf2, which belongs to the cap “n” collar family of basic region-leucine zipper (bZIP) transcription factors, plays a central role in the regulation of basal and inducible expression of genes encoding phase II cytoprotective enzymes. Nrf2 has six highly conserved protein regions called Nrf2-ECH domains, Neh1 to Neh6. At its C terminus, Nrf2 has a bZIP structure enabling dimerization with small musculoaponeurotic fibrosarcoma virus (Maf) proteins and for binding to the ARE. At its N terminus, Nrf2 has a Neh2 domain (amino acid residues 1–95) that is highly conserved between species [24]. Neh3–5 contains residues important for Nrf2 transcriptional activity. Neh6 functions as a degron and mediates nuclear Nrf2 degradation [25]. In the absence of oxidative stress, Nrf2 pathway activity is suppressed by the sequestration and degradation of Nrf2 in the cytoplasm. However, in the presence of oxidative stress, Nrf2 translocates to the nucleus.

Keap1 is a cysteine-rich negative regulator of Nrf2. McMahon et al. reported that Keap1 controlled the total cellular level of Nrf2 protein in the RL34 nontransformed rat liver cell line. In the absence of oxidative stress, Nrf2 interacts with Keap1 and induces more rapid proteasomal degradation than that observed in the presence of oxidative stress. Keap1 negatively regulates Nrf2 function by controlling its subcellular location. Specifically, Keap1 sequesters the bZIP protein of Nrf2 in the cytoplasm, leading to low expression of ARE-driven genes. During oxidative stress, a signal that involves phosphorylation and/or redox modification is transduced to the Keap1-Nrf2 complex leading to its disruption, facilitating nuclear translocation of Nrf2 [26].

The Keap1 protein consists of distinct structural domains including the N terminal Broad complex, Tramtrack, and bric-a-brac (BTB) domains, the central intervening region (IVR), the double glycine repeat (DGR) domain where Nrf2 binding occurs, and the C terminal region [27]. Three important cysteine residues have been identified within Keap1: C151, within the BTB domain, and C273 and C288, within the IVR. C273 and C288 coordinate a zinc ion and are crucial for maintaining the structural integrity required for Keap1 to associate with Nrf2. It is believed that oxidative modification of these two cysteine residues releases the zinc ion and, consequently, Nrf2 ubiquitination is abolished [28].

**Figure 2:**
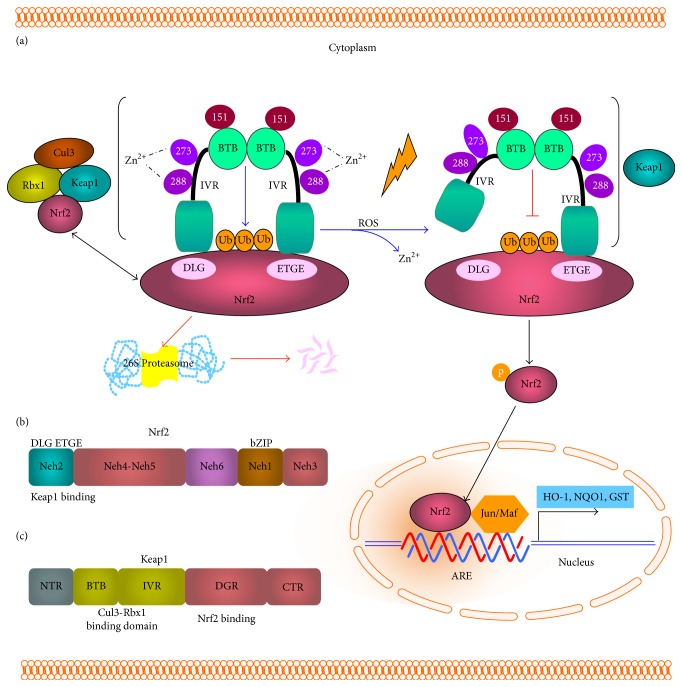
The Kelch-like chicken erythroid-derived cap “n” collar homology factor-associated protein 1- (Keap1-) NF-E2-related factor 2- (Nrf2) antioxidant response element (ARE) signaling pathway. (a) Keap1-Nrf2 interactions are mediated via the high affinity ETGE motif “hinge” site and the lower affinity DLG motif “latch” site within the Nrf2 Neh2 domain. Under normal cellular conditions, Nrf2 first interacts with the Keap1 dimer through the ETGE hinge and subsequently with the cullin-3- (Cul3-) ring box 1 (Rbx1) complex via the DLG latch motif, which leads to the ubiquitination and degradation of Nrf2. During cellular stress, the hinge and the latch of Nrf2 may be disrupted by changes to Keap1 homodimer formation and translocation to nucleus. (b) The structure of Nrf2, including Neh1–6 domains. (c) Keap1 is composed of distinct structural domains, including the N terminal region (NTR), Broad complex, Tramtrack, and bric-a-brac (BTB) domain, intervening region (IVR), double glycine repeat (DGR; Nrf2 binding region), and C terminal region (CTR).

Keap1-Nrf2 interactions are mediated via the high affinity ETGE motif “hinge” site within the Nrf2 Neh2 domain and the lower affinity DLG motif “latch” site. Under normal cellular conditions, Nrf2 first interacts with the Keap1 dimer through the ETGE hinge and subsequently with the cullin-3- (Cul3) ring box 1 (Rbx1) complex via the DLG latch motif, which leads to the ubiquitination and degradation of Nrf2. During cellular stress or pharmacologic induction, the hinge and the latch of Nrf2 may be disrupted by changes to Keap1 homodimer formation [49] (Figure 2). The available evidence indicates that Nrf2 activators (such as SFN) may block Nrf2 ubiquitination by altering Keap1 conformation by interacting with the thiol groups of specific cysteine residues within Keap1 [49].

The ARE is a* cis*-acting enhancer sequence, also referred to as the electrophile response element (EpRE), with a core nucleotide sequence of 5′-RTGACNNNGC-3′; this element regulates the basal and inducible expression of more than 200 genes [50]. Transcriptional activation of ARE-mediated genes requires Nrf2 heterodimerization with other bZIP proteins, including Jun (c-Jun, Jun-D, and Jun-B) and small Maf (MafG, MafK, and MafF) proteins. In addition, the Nrf2 gene contains two ARE-like promoter sequences, indicating that Nrf2 autoregulation could sustain ARE-mediated gene expression [51]. Furthermore, ARE inducers can inactivate Keap1 by inducing a conformational rearrangement of this protein; this facilitates Nrf2 nuclear translocation and further activation of ARE-mediated transcription of protective genes, in association with bZIP proteins.

### 3.2. Nrf2 Antioxidant Mechanism

Nrf2 plays pivotal roles in coordinating the antioxidant response and maintaining redox homeostasis. Nrf2 expression is normally suppressed but is strikingly induced under conditions of oxidative stress. Li et al. provided a mechanistic explanation for the translational control of Nrf2 by oxidative stress [52]. An internal-ribosomal-entry site (IRES) was identified within the 5′-untranslated region of human Nrf2 mRNA. The Nrf2-IRES contains a highly conserved 18S rRNA binding site that is required for internal initiation. This Nrf2-IRES also contains a hairpin structured inhibitory element located upstream of the rRNA binding site. Deletion of this inhibitory element remarkably enhanced translation. Significantly, treatment of cells with hydrogen peroxide and SFN further stimulated IRES-mediated Nrf2 translation [52]. Thimmulappa et al. also elucidated another mechanism underlying the translational control of Nrf2 by comparing the transcriptional profiles of small intestines from wild-type (Nrf2^+/+^) and knock-out (Nrf2^−/−^) mice treated orally with vehicle or SFN (9 *μ*M/day) for 1 week. This study identified numerous genes regulated by Nrf2, including previously reported Nrf2-regulated genes (NQO1, GST, UDP-glucuronosyltransferases, and epoxide hydrolase) and a number of genes that were not previously known to be regulated by Nrf2 [53]. Furthermore, Nioi et al. characterized the NQO1 5′-upstream region in murine Nrf2^+/+^ and Nrf2^−/−^ embryonic fibroblasts and showed that Nrf2 was essential for both the constitutive expression of NQO1 and its induction by SFN. Electrophoretic mobility-shift and chromatin immunoprecipitation assays revealed that low levels of Nrf2 were constitutively associated with the NQO1 ARE; following exposure to SFN, substantially greater quantities of Nrf2 were recruited to the ARE as a heterodimer with the small Maf protein [54]. These findings demonstrated the key role played by Nrf2 in mediating detoxification and/or antioxidant effects, thereby protecting cells from genotoxic damage.

### 3.3. Nrf2 Nuclear Localization

Nrf2 is the key transcription factor regulating the antioxidant response and its site of action is within the nucleus. Li et al. identified a nuclear localization signal (NLS) within the basic region of Nrf2 and a nuclear exporting signal (NES) within the bZIP domain [55]. They also characterized a new functional NES within the transactivation (TA) domain of Nrf2. A variety of oxidants, including SFN, effectively induced nuclear translocation of a green fluorescent protein-tagged Nrf2 segment. The discovery of multiple NLS/NES motifs in Nrf2 and the redox sensitivity of NESTA imply that Nrf2 may transduce oxidative signals into the nucleus, consequently initiating antioxidant gene transcription [55]. Nrf2 is known to promote the transcription of many cytoprotective genes and is a major drug target for the prevention of cancers and other diseases. Li et al. demonstrated that while the activation of Nrf2 by prototypical chemical activators, including SFN, was mediated solely by inhibition of its ubiquitination, such inhibition occurred predominantly in the nucleus [56]. Pickering et al. further proved that increasing the cellular capacity to degrade oxidized proteins required increased cellular levels of Nrf2, translocation of Nrf2 from the cytoplasm to the nucleus, and maximal increases in proteolytic capacity [57].

We have previously employed immunofluorescent staining to identify SFN-stimulated nuclear translocation of Nrf2 in diabetic cardiomyopathy (DCM), resulting in the upregulation of the expression of Nrf2 and its downstream genes [47]. Exogenous chemical activators of Nrf2 have often been suggested to inhibit Nrf2 ubiquitination and degradation, thereby increasing its nuclear translocation [56]. These findings provided significant new insights into Nrf2 activation. On the other hand, the oxidative stress or antioxidants can also induce the expression of intracellular kinases, such as mitogen-activated protein kinase (MAPK), phosphatidylinositol 3-kinase (PI3K), and protein kinase C (PKC); as a result, these kinases phosphorylate Nrf2 to produce p-Nrf2, affecting nuclear-cytoplasmic trafficking of Nrf2 or modulating Nrf2 protein stability [58]. Therefore, the ability to adapt to acute oxidative stress by interrupting the cytoplasmic degradation of Nrf2 and promoting its nuclear translocation is an important component of cellular defense mechanisms.

In summary, the Nrf2 Neh2 domain binds with the region between the BTB and DGR Kelch repeat domains of actin-bound Keap1. Exposure of cells to ARE inducers results in the dissociation of Nrf2 from Keap1 and facilitates translocation of Nrf2 to the nucleus, where it heterodimerizes with a small Maf protein and binds to ARE, resulting in the transcriptional activation of target genes [59]. Therefore, the Keap1-Nrf2-ARE pathway represents one of the most important cellular defense mechanisms against oxidative stress and xenobiotic damage. Transcriptional activation of ARE-regulated phase II detoxification and antioxidant genes by Nrf2 is considered to represent the prime mechanism involved in the antioxidant effects of SFN.

## 4. SFN-Mediated Activation of Nrf2 Signal Transduction Pathways

### 4.1. Direct Effects of SFN on Nrf2 via Related Signaling Pathways

As SFN induces Nrf2 accumulation due to inhibition of proteasomal degradation of the bZIP protein [26], it has attracted interest for the promotion of human health and wellbeing [12]. SFN is an electrophile that can react with protein thiols to form thionoacyl adducts; this is believed to affect the critical Keap1 cysteine residues and block Nrf2 polyubiquitination and degradation, resulting in Nrf2-Keap1-ARE signaling and translocation Nrf2 to nucleus. Our research group also found that SFN induced significantly higher Nrf2 levels expression levels in the cardiac cell nuclei [47].

### 4.2. Indirect Effects of SFN on Nrf2 via Related Signaling Pathways

**Figure 3:**
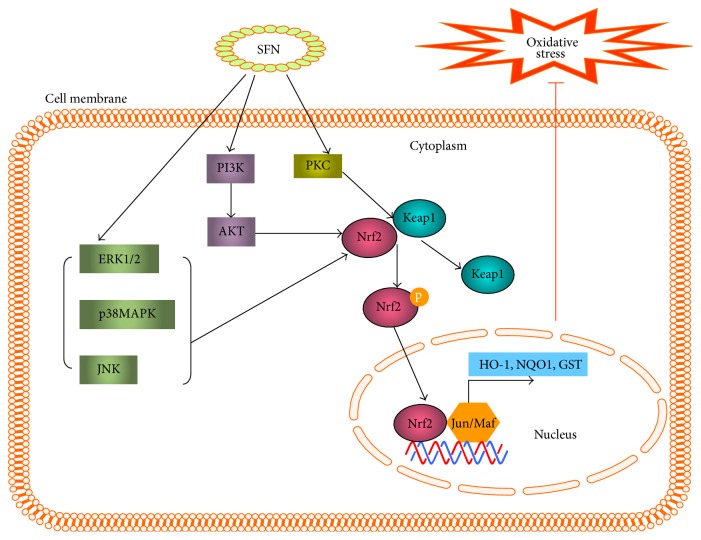
Sulforaphane (SFN) activation of Nrf2 signaling. SFN activates three mitogen-activated protein kinases (MAPKs): extracellular signal-regulated protein kinase (ERK), c-Jun N-terminal kinase (JNK), and p38; these may stimulate Nrf2 activation. SFN may also activate protein kinase C (PKC), which directly phosphorylates Nrf2. Alternatively SFN activates Nrf2 through phosphatidylinositol 3-kinase (PI3K) and protein kinase B (AKT). Ultimately, Nrf2 phosphorylation triggers nuclear translocation and induction of antioxidant-response element- (ARE-) mediated gene transcription, reducing oxidative stress.

The Nrf2-Keap1-ARE signaling pathway can be modulated by several upstream kinases including MAPK, PI3K, and PKC [60]. SFN may affect the activity of a variety of intracellular kinases that phosphorylate Nrf2 to produce p-Nrf2, altering nuclear-cytoplasmic trafficking of Nrf2 or modulating Nrf2 protein stability [59, 60]. Previous studies have reported that Nrf2 activation is potentiated by its phosphorylation of Nrf2 serine 40 [15]. Leoncini et al. used cultured rat cardiomyocytes to analyze the signaling pathways involved in the protective effects of SFN. This study investigated the ability of SFN to activate protein kinase B (AKT) and extracellular signal-regulated kinases 1 and 2 signaling pathways; these are implicated in cardiac cell survival and also increase Nrf2 phosphorylation and ARE binding. Using specific inhibitors, this study demonstrated that the PI3K/AKT pathway mediated the influence of SFN on the expression and activity of GR, GST, Trx reductase, and NQO1 (analyzed by western blot and spectrophotometric assay, resp.); these effects modulated Nrf2 binding and phosphorylation, resulting in a cytoprotective effect against oxidative damage [61] (Figure 3).

### 4.3. Long-Term SFN-Mediated Effects

Although absorbed SFN is cleared from the body within a few hours, some findings demonstrated that ingestion of broccoli or SFN induced long-term protection against free radical-induced damage. In order to explore the cause of this phenomenon, Bergström et al. studied the kinetics of the SFN-induced Nrf2 response in astrocytes, which is a cell type known to be highly involved in the defense against oxidative stress in the brain [62]. The results of this analysis showed that 4 h SFN exposure induced Nrf2-dependent increases in NQO1 and HO-1 mRNAs that persisted for 24 h, and the levels of the corresponding proteins remained elevated for over 48 h. In addition, peroxide-clearing activity and glutathione levels were elevated for more than 20 h, resulting in an increased resistance to superoxide-induced cell damage [62]. In cultured rat neonatal cardiomyocytes, Angeloni et al. also showed that SFN induced long-term decreases in intracellular ROS production, increased cell viability, and decreased DNA fragmentation, accompanied by the induction of antioxidants and phase II enzymes [63]. Our research group has shown that diabetic mice treated with SFN for three months showed a reduced incidence of DCM at the end of the treatment (3 months) and also after 6 months [47]. These findings indicate that short-term stimulation of the Nrf2 pathway by SFN results in a long-term elevation of endogenous antioxidant effects.

Taken together, these reports indicate that SFN can induce phase II enzyme expression via either direct or indirect activation of Nrf2-Keap1-ARE signaling, which is very important for our understanding of the mechanisms underlying the effects of SFN on Nrf2 activity.

## 5. SFN-Mediated Protection from Hypertension

The prevalence of hypertension increases markedly with age. It is one of the major causes of CVD, and prevention of hypertension may play a crucial role in protesting against CVD. Oxidative stress caused by reduced production of nitric oxide and/or increased production of ROS (mainly superoxide) may promote endothelial dysfunction. Therefore, increased oxidative stress represents one possible driver of the increased prevalence of hypertension [64]. Noyan-Ashraf et al. demonstrated that a diet containing broccoli sprouts high in Grn (Grn+) decreased oxidative stress and associated problems in male spontaneously hypertensive stroke-prone rats (SHRsp). As one of Grn key metabolites, SFN was also found to improve blood pressure [30].

Females on a Grn+ diet also showed decreased oxidative stress and associated problems, such as hypertension, as compared with females administered a control diet. Oxidative stress in pregnant female rats can affect fetal determinants of adult health and the offspring of pregnant females administered the Grn+ diet also had lower blood pressure and less tissue inflammation in adulthood; regardless of their subsequent diet, offspring of mothers fed a Grn+ diet had the best health outcomes [29].

Regarding the mechanism underlying SFN-mediated protection from hypertension, there is evidence that SFN-induced upregulation of phase II enzymes reduces the oxidative stress experienced by the vascular smooth muscle cell (VSMC), particularly in SHR. Wu and Juurlink found that, in the pathogenesis of hypertension, basal levels of cellular GSH, GR, and glutathione peroxidase (GPX) were significantly lower in SMC from SHRsp than in those from normotensive Wistar-Kyoto rats. SFN (0.05–1 *μ*M) induced significant and concentration-dependent increases in the levels of GSH and HO-1 protein, in the activities of GR and GPX, and in VSMC from both rat strains [32]. In a subsequent study, Wu et al. further demonstrated that SFN significantly increased GSH content, decreased levels of oxidized GSH, decreased protein nitrosylation, and increased activities of GR and GPX; these changes correlated with improved endothelial-dependent relaxation of the aorta and significantly lower blood pressure [33]. Although these studies did not directly measure Nrf2 expression and function, the observed effects were most likely due to the upregulation of Nrf2 expression and function induced by SFN.

## 6. SFN-Mediated Protection from Atherosclerosis 

Atherosclerosis is found in a range of CVD, including myocardial infarction, stroke, and peripheral artery disease; these remain major causes of morbidity and mortality globally [65, 66]. It is generally accepted that shear stress plays a key role in the development of early atherosclerosis [67]. Atherosclerotic lesions are preferentially located at the outer walls of the arterial branches and curvatures, where the local flow is disturbed; steady laminar blood flow (s-flow) and sustained high shear stress can modulate the expression of genes and proteins that protect against atherosclerosis in endothelial cells (EC) [68]. Dekker et al. identified Krüppel-like factor 2 (KLF2) as a transcription factor specifically induced by s-flow in EC. Nrf2 was identified as a shear-induced transcription factor that is responsible for antioxidant gene expression. It was also shown that both of these transcription factors were induced in s-flow regions of the vasculature* in vivo*. KLF2 increased the nuclear localization of Nrf2 and the combined actions of these two factors constituted about 70% of the s-flow-induced endothelial gene expression [68]. Zakkar et al. also investigated whether Nrf2 regulated arterial EC activation. En face staining revealed that Nrf2 was activated in EC at an atheroprotected region of the murine aorta but was present in an inactive form in EC at an atherosusceptible site. Treatment with SFN activated Nrf2 at the susceptible site in wild-type but not Nrf2^−/−^ animals, indicating that it suppressed EC activation via Nrf2 [35].

Atherosclerosis is also associated with long-term inflammatory disease of the arterial wall. Increased expression of cell adhesion molecules such as intercellular adhesion molecule-1 (ICAM-1) and vascular cell adhesion molecule-1 (VCAM-1) is associated with increased proliferation of VSMC, leading to increased neointimal or atherosclerotic lesion formation. Kim et al. investigated the effect of SFN on the expression of VCAM-1 in cultured mouse VSMC. Pretreatment of VSMC for 2 h with SFN (1–5 *μ*g/mL) dose-dependently inhibited TNF-*α*-induced protein expression of VCAM-1. SFN also suppressed TNF-*α*-induced production of intracellular ROS [34]. Kwon et al. further investigated the effects of SFN on VSMC proliferation and neointimal formation in a carotid artery injury model and found that SFN inhibited TNF-*α*-induced VCAM-1 mRNA and protein expression in VSMC [36]. These* in vivo* and* in vitro* animal studies indicated that SFN had beneficial effects, suppressing inflammation within the atherosclerotic lesion. To investigate the effects of SFN on endothelial inflammatory gene expression in human aortic EC (HAEC), Chen et al. demonstrated that TNF-*α*-induced VCAM-1 mRNA and protein levels in HAEC were suppressed after treatment of HAEC with SFN (1–4 *μ*M) for 1 h, which suggested that SFN may be useful as a therapeutic agent for the treatment of inflammatory diseases [37].

In addition to these inflammatory mechanisms, endothelial lipase (EL) is also involved in atherosclerosis-EL is a member of the triacylglycerol lipase family and it decreases the levels of high-density lipoprotein (HDL) cholesterol. Kivelä et al. demonstrated that SFN inhibited TNF-*α*-mediated induction of EL in human umbilical vein EC (HUVEC), indicating that SFN may have a beneficial effect on HDL cholesterol levels; this would reduce the risk for atherosclerosis [38]. Oxidized low-density lipoprotein (oxLDL) contributes to atherogenesis through multiple mechanisms. Huang et al. reported that treatment with SFN (0–10 *μ*M) reversed oxLDL-induced ROS production by inducing dose-dependent increases in HO-1 levels, expression of GCL catalytic and modifier subunits, intracellular glutathione content, and ARE-luciferase reporter activity [69]. Collectively, these results indicated that SFN modified inflammation, EL activity, and oxLDL levels, all of which play important roles in atherosclerosis.

Obesity is associated with metabolic disorder, which is another risk factor for atherosclerosis and CVD. Choi et al. investigated whether SFN prevented high-fat diet- (HFD-) induced obesity in C57BL/6N mice [70]. Mice were fed with a normal diet (ND), HFD, or HFD plus 0.1% SFN for 6 weeks. Food efficiency ratios and body weights were lower in the HFD-SFN-fed mice than in the HFD mice. SFN attenuated HFD-induced visceral adiposity, adipocyte hypertrophy, and fat accumulation in the liver. Serum total cholesterol and leptin and liver triglyceride levels were lower in HFD-SFN mice than in HFD mice. These results suggested that SFN may induce antiobesity effects by inhibiting adipogenesis [70].

Platelet aggregation plays an important role in arterial thrombosis, which leads to atherosclerosis and CVD. Chuang et al. proved that SFN inhibited human platelet aggregation and reduced thrombus formation under flow conditions [71]. Oh et al. also found that SFN inhibited platelet aggregation dose-dependently. A comparison of platelet activators revealed that collagen was most inhibited by SFN, which markedly decreased collagen-induced glycoprotein IIb/IIIa activation and thromboxane A2 formation* in vitro*. SFN also reduced collagen and epinephrine-induced pulmonary embolism but did not affect prothrombin time* in vivo*; this indicated that SFN might be a potential antithrombotic drug [72, 73].

Taken together, the above animal and human studies indicate that SFN-medicated activation of Nrf2 reduces EC activation at atherosusceptible sites, regulates migration and proliferation of VSMC, decreases the expression of inflammatory factors, prevents HFD-induced obesity, and has antithrombic effect. These findings indicate that SFN may provide a potential therapeutic agent for the prevention of atherosclerosis, thereby reducing the risk for CVD.

## 7. SFN Protects from Cardiac Ischemia-Reperfusion (I/R) Injury

It has been demonstrated that I/R injury is crucial in the pathology of major CVD, such as stroke and myocardial infarction [74]. Mukherjee et al. [10, 75] fed a group of rats with broccoli by oral gavage. After 30 days, the rats were sacrificed and their isolated perfused hearts were made ischemic for 30 min, followed by 2 h reperfusion. Treatment with broccoli provided significant cardioprotection, as evidenced by improved postischemic ventricular function, reduced myocardial infarct size, and decreased cardiomyocyte apoptosis along with significant reduction of cytochrome c release and procaspase 3 activity. These protective effects were found to be associated with the enhanced activity of Nrf2 [10, 75]. Similarly, subsequent studies showed that SFN protected against cardiac I/R injury. They found that SFN treatment attenuated I/R-induced decreases in Mn-superoxide dismutase, catalase, and HO-1 levels [39, 76]. The protective effects of SFN against I/R injury may be partly mediated through the Nrf2-Keap1-ARE antioxidant pathway.

## 8. SFN Protects from Diabetes and Diabetes-Induced Complications 

### 8.1. SFN Protects from Diabetes

Diabetes has reached epidemic levels worldwide. Increased levels of ROS, mainly produced by the NADPH oxidase family, have been identified in the pathogenesis of experimental and clinical diabetes [77, 78]. Patients with diabetes are two to four times more likely to develop CVD than those without diabetes; indeed CVD is the leading cause of premature death and disability in patients with diabetes [79]. de Souza et al. evaluated the effect of SFN in streptozotocin- (STZ-) induced diabetes. Male Wistar rats were given water or 0.1, 0.25, or 0.5 mg/kg SFN by oral gavage before injection of STZ (80 mg/kg). Diabetic animals had elevated levels of serum triacylglycerols, urea, and creatinine, and all SFN doses were able to reverse these alterations [40]. Pretreatment with SFN also blocked the development of type 1 diabetes in STZ-treated mice and inhibited cytokine-induced *β*-cell damage [41].

SFN protection against oxidative stress-induced *β*-cell damage or death was also observed under other experimental conditions. For instance, islet cell transplantation is often limited by poor transplanted *β*-cell survival. SFN was found to significantly prolong the survival time of these cells by protecting them against transplantation-induced oxidative damage [80]. Based on the above studies, we can conclude that SFN had protective effects on the pancreas and this may have a potential clinical application in improving the condition of this organ in hemodynamically unstable donors.

In view of the fact that the etiologic factors involved in the development of type 2 diabetes are complicated and multifaceted, including obesity, systemic chronic inflammation, hepatic steatosis, hyperlipidemia, and insulin resistance, Xu et al. have investigated the role of Nrf2 in the prevention of hepatic steatosis, insulin resistance, obesity, and type 2 diabetes. Obesity and lipid accumulation in white adipose tissue were significantly decreased in HFD-treated Keap1-knockdown mice, which showed increased Nrf2 expression and function, as compared with C57Bl/6J wild-type mice [81]. In addition, compared with the Lep(ob/ob) mice, a common model of type 2 diabetes model, Lep(ob/ob)-Keap1-knockdown mice had increased Nrf2 activity and decreased lipid accumulation, smaller adipocytes, and reduced lipogenic gene expression [81]. Adipocyte differentiation is triggered by adipocyte hyperplasia, which leads to obesity. Choi et al. [31] demonstrated that SFN decreased the accumulation of lipid droplets and triglycerides in these adipocytes in 3T3-L1 cells and also inhibited the proliferation of adipocytes. These results suggested that insulin resistance, metabolic syndrome, and diabetes could be prevented by SFN-mediated effects on Nrf2 expression and function.

### 8.2. SFN Protects against Diabetes-Induced Complications

Diabetes complications are associated with micro- and macrovascular complications, and the major complications include nephropathy, retinopathy, neuropathy, DCM, and accelerated atherosclerosis. Currently, high glucose-driven production of ROS intermediates and inflammatory damage are recognized contributors to complications of diabetes. A systemic review stated that supplementation with broccoli sprouts containing high levels of SFN increased plasma total antioxidant capacity and decreased the oxidative stress index, lipid peroxidation, serum triglycerides, oxLDL/LDL-cholesterol ratio, serum insulin, insulin resistance, and serum high-sensitive C-reactive protein* in vivo.* [82]. The pathological state is defined as metabolic syndrome, which is a risk factor for diabetes and the complications of diabetic cardiac-cerebral vascular disease. Therefore, alleviation of oxidative and inflammation status may protect from diabetes and diabetes-induced complications.

Diabetic nephropathy is the most common cause of end-stage kidney disease worldwide and is associated with increased morbidity and mortality in patients with both type 1 and type 2 diabetes [83]. Diabetic nephropathy results in premature death from accelerated CVD and various other complications [79]. It was also shown that the dual antioxidant and anti-inflammatory activities of SFN effectively activated Nrf2 and reversed the various deficits observed in experimental diabetic neuropathy [44]. Zheng et al. studied Nrf2^+/+^ and Nrf2^−/−^ with STZ-induced diabetes. SFN was administered 2 weeks after STZ injection and metabolic indices and renal structure and function were assessed at 18 weeks. SFN significantly attenuated common symptoms of metabolic disorder that are associated with diabetes in Nrf2^+/+^ mice but not in Nrf2^−/−^ mice, indicating that SFN operated via specific activation of the Nrf2 pathway. Furthermore, SFN improved renal performance and minimized pathological changes in the glomeruli of STZ-Nrf2^+/+^ mice [42]. Cui et al. also demonstrated that SFN injection (0.5 mg/kg daily for 3 months) prevented diabetic nephropathy in a type 1 diabetic mouse model induced by multiple low doses of STZ [43].

Xue et al. assessed whether activation of Nrf2 by SFN in human microvascular EC (HMEC-1) prevented metabolic dysfunction in hyperglycemia. HMEC-1 cells were incubated with media containing low and high glucose concentrations (5 and 30 mM, resp.). SFN induced nuclear translocation of Nrf2 and increased ARE-linked gene expression. Hyperglycemia increased the formation of ROS, an effect that was linked to mitochondrial dysfunction and prevented by SFN [45]. Miao et al. demonstrated significant increases in oxidative stress and inflammation in the aorta in a type 1 STZ-induced diabetic model at both 3 and 6 months, with fibrotic responses after 6 months of diabetes. SFN completely prevented these pathogenic diabetic changes and also significantly upregulated the expression of Nrf2 and its downstream antioxidants [46].

Diabetes-induced cardiomyopathy is one of the major types of CVD. Our research group investigated whether SFN prevented DCM. Type 1 diabetes was induced in FVB mice by multiple intraperitoneal injections of low doses of STZ. Hyperglycemic and age-matched control mice were treated with or without 0.5 mg/kg SFN (Sigma, L-SFN, 2.8 *μ*mol/kg) daily, five days/week for 3 months, and then kept until 6 months after the induction of diabetes. At 3 and 6 months of diabetes, SFN significantly prevented diabetes-induced hypertension and cardiac dysfunction and also prevented diabetes-induced cardiac hypertrophy and fibrosis. SFN almost completely prevented diabetes-induced cardiac oxidative damage and inflammation. SFN upregulated Nrf2 expression and transcriptional activity, as evidenced by increased Nrf2 nuclear accumulation and phosphorylation, as well as increased mRNA and protein expression of Nrf2 downstream antioxidants. These results suggested that DCM could be prevented by SFN via upregulation of Nrf2 expression and transcriptional activity [47]. Zhang et al. investigated the effects of SFN on type 2 diabetes-induced cardiac lipid accumulation and, subsequently, DCM. SFN treatment significantly attenuated cardiac remodeling and dysfunction, significantly inhibited cardiac lipid accumulation as measured by Oil Red O staining, and improved cardiac inflammation, oxidative stress, and fibrosis [48].

Based on these improvements, SFN-induced activation of Nrf2 counteracted glucose-induced ROS and inflammation, protecting against diabetes and diabetic complications.

## 9. Prospects on the Clinical Application of SFN in the Prevention of CVD

It has been shown that SFN produced protective effects against CVD* in vitro* and animal studies [29, 34, 47, 75]. However, there are no systematic clinical studies in identifying the optimal consumption level of SFN in humans. Herein, based on the current results of the studies, the dose and application form of SFN and its rationalities are discussed to provide basis for further CVD prevention clinical trials.

Since SFN can be found in brassicates like broccoli, it will be reasonable to have a diet containing broccoli to prevent CVD in the studied group. But what amounts of SFN are contained in the brassicates? Saha et al. reported that extraction of 100–200 mg broccoli powder using a series of purification steps provided a reasonable dose of SFN (23.5 *μ*mol) [84]. Cramer et al. showed that incubation in water at room temperature for 24 h with the addition of 0.8 U myrosinase fresh broccoli sprouts produced 1.69 *μ*mol SFN/g fresh weight on average, as determined by bench hydrolysis [85].

As these were taken orally, the dose of SFN was also influenced by its bioavailability in the body. SFN bioavailability from dietary sources is a critical determinant of its efficacy in humans. Atwell et al. evaluated SFN absorption from a myrosinase-treated broccoli sprout extract. Subjects consumed fresh broccoli sprouts or the extract, each providing 200 *μ*mol SFN daily, as a single dose and as two 100 *μ*mol doses taken 12 h apart. The study showed that 12 h dosing was associated with higher plasma SFN metabolite levels at later time points than those achieved by 24 h dosing. This indicated that the dietary form and dosing schedule of SFN may influence its absorption and efficacy in human [86].

Then, what amount of SFN should be suggested to consume in order to achieve the levels for prevention of CVD? It has shown that 10 *μ*M SFN can protect cardiomyocytes* in vitro* and that 0.5 mg/kg (2.8 *μ*mol/kg) SFN could prevent CVD in STZ induced diabetes mice. According to the conversion formula between mice and human [87, 88], the consumption of SFN in human is supposed to be 0.05 mg/kg (0.28 *μ*mol/kg) per day. That is, if a man is with the body weight of 70 kg, the dose of SFN will be 19.6 *μ*mol per day, and the consumption of fresh broccoli dose will be about 12 g per day. It is much lower than the dose (150 *μ*mol per day [89]) used in the cancer chemoprevention trial without showing any toxic effects [90]. Therefore, it implies that SFN will be much well-tolerated if it is used clinically in the prevention of CVD.

Collectively, a diet containing SFN, such as brassicates, could prevent CVD, and the required consumption of SFN is easy to get and shows harmless. It is worthwhile to perform clinical trials to study the effects and optimal dose of SFN in prevention of CVD.

## 10. Conclusions 

Oxidative stress plays a major role in the pathophysiology of cardiac disorders. SFN found in cruciferous vegetables is an indirect antioxidant that can activate Nrf2 and its downstream target genes to induce antioxidant effects. Animal and human experiments have identified substantial SFN-mediated protection from a range of CVD, including hypertension, atherosclerosis, I/R injury, diabetes, and diabetic complications (Table 1). The findings presented in this review indicate that SFN, a phytochemical isolated from extracts of an edible plant with a presumed low level of toxicity, protects against CVD. SFN could therefore contribute to the prevention of CVD.

## Figures and Tables

**Table 1:** Summary of *in  vivo* and *in  vitro* studies of sulforaphane- (SFN-) mediated protection against cardiovascular disease (CVD).

CVD	Model	SFN dose	Biomarker modulation	Reference
Hypertension	Female SHRsp rats on the Grn+ and Grn diets (1st generation), and the offspring (2nd generation)	0.9 mg SFN in 200 mg air-dried broccoli sprouts	Female SHRsp on a Grn+ diet had decreased hypertension. Their offspring had lower blood pressure in adulthood, regardless of diet, and the best health outcomes	[29]
	Female SHRsp rats, oral gavage of SFN in corn oil	1.77 mg/kg	Mean arterial pressure was 20% higher in vehicle-treated SHRsp and SFN administration to SHRsp improved blood pressure	[30]
	3T3-L1 preadipocytes, cultured with SFN	20 *μ*M for 6 days	SFN inhibited early-stage adipocyte differentiation	[31]
	Isolated aortic SMCs from SHRsp rats or controls	0.05–1 *μ*M for 24 h	SFN induced concentration-dependent increases in cellular GSH levels and HO-1 protein content and decreased oxidative stress	[32]
	Male SHRsp and Sprague-Dawley rats on control, Grn+, and Grn− diets	0.9 mg SFN in 200 mg air-dried broccoli sprouts	SHRsp fed a Grn+ diet had a major improvement in the cardiovascular and kidney tissues and reduced hypertension	[33]

Atherosclerosis	Mouse VSMCs incubated with TNF-*α* (10 ng/mL) for 4 and 8 h	Pretreatment with 5, 15, and 25 *μ*M for 2 h	SFN dose-dependently inhibited TNF-*α*-induced protein expression of VCAM-1 and intracellular ROS, which may have beneficial effects on inflammation within the atherosclerotic lesion	[34]
	Male C57BL/6 or Nrf2^−/−^ mice aortic EC were stained at susceptible and protected sites	Pretreatment with 5 mg/kg for 4 h or 24 h	SFN activation of Nrf2 reduced endothelial activation at atherosusceptible sites	[35]
	Confluent HUVEC exposed to unidirectional laminar shear for 24 h	1 *μ*M, 24 h	SFN inhibited TNF-*α*-induced VCAM-1 expression and prevented VSMC proliferation	
	Sprague-Dawley rats subjected to carotid artery balloon injury; VSMCs were exposed to TNF-*α*	5 *μ*M	SFN attenuated neointima formation after balloon injury and intima area ratio and stenosis	[36]
	HAEC treated with TNF-*α* (100 U/mL) for 4 h	1–4 *μ*M pretreatment of HAECs for 1 h	SFN suppressed TNF-*α*-induced VCAM-1 protein expression, suggesting that SFN may be a useful treatment for inflammatory diseases	[37]
	HUVEC treated with 0–40 ng/mL TNF-*α* for 24 h or with 10 ng/mL TNF-*α* for 0–48 h	10 *μ*M pretreatment for 1 h	SFN inhibited TNF-*α*-mediated induction of endothelial lipase in HUVEC, indicating that SFN may have a beneficial effect on HDL cholesterol levels	[38]

I/R	Ischemia in isolated perfused Langendorff Sprague-Dawley rat hearts	0.5 mg/kg daily i.p. pretreatment for 3 days before ischemia	SFN significantly improved coronary flow and reduced I/R-induced increases in LDH level and infarct size, showing that SFN protected against I/R injury	[39]

Diabetes	Male Wistar rats injected with STZ (80 mg/kg)	Oral pretreatment with 0.1, 0.25, or 0.5 mg/kg	All SFN doses reduced levels of triacylglycerol, urea, and creatinine, cholesterol, alanine, and aspartate aminotransferase levels	[40]
	RIN cells treated with IL-1*β* (2 ng/mL) and IFN-*γ* (100 U/mL) to produce H_2_O_2_	2.5–10 *μ*M pretreatment for 3 h	Pretreatment with SFN resulted in concentration-dependent protection against the toxic effect of cytokines, with increased survival of RIN cells	[41]

Diabetic nephropathy	Eight-week-old mice received STZ (50 mg/kg) i.p. for 5 consecutive days	Pretreatment with 12.5 mg/kg i.p. three times a week for 16 weeks	SFN significantly attenuated common metabolic disorder symptoms, improved renal performance, and minimized pathological alterations in the glomerulus of STZ-Nrf2^+/+^ mice	[42]
	Mice were injected i.p. with 50 mg/kg STZ daily for 5 days	Subcutaneous injection of 0.5 mg/kg, five days per week, for 3 months	SFN prevented diabetes-induced renal inflammation and oxidative stress and also prevented renal structural changes and fibrosis	[43]

Diabetic neuropathy	Diabetic neuropathy was induced in rats using STZ	Administration of 0.5 and 1 mg/kg six weeks after diabetes	SFN reduced NF-*κ*B expression and I*κ*B kinase phosphorylation, along with abrogation of inducible nitric oxide synthase, cyclooxygenase-2 expression, and TNF-*α* and IL-6 levels	[44]

Diabetic angiopathy	HMEC-1 cultured in high glucose medium (30 mM)	4 *μ*M for 6–48 h	Multiple pathways of biochemical dysfunction in HMEC-1 cells induced by hyperglycemia were reversed by SFN	[45]

DCM	Mice were injected with 50 mg/kg STZ i.p. daily for 5 days	0.5 mg/kg subcutaneous injection daily for 3 months after STZ-induced diabetes	Diabetes induced significant increases in oxidative stress and inflammation in the aorta at both 3 and 6 months, and fibrotic response at 6 months. SFN completely prevented these pathogenic changes	[46]
	Mice were injected with 50 mg/kg STZ i.p. daily for 5 days	0.5 mg/kg subcutaneous injection daily for 3 months after STZ-induced diabetes	SFN significantly reduced hypertension and cardiac dysfunction at both 3 and 6 months and also prevented cardiac hypertrophy and fibrosis. SFN also almost completely prevented cardiac oxidative damage and inflammation	[47]
	Mice were fed a high-fat diet for 3 months, then treated with 100 mg/kg STZ i.p. to induce T2DM	0.5 mg/kg subcutaneous injection daily five days a week for 4 months	SFN significantly inhibited cardiac lipid accumulation improved cardiac inflammation oxidative stress and fibrosis induced by T2DM	[48]

Notes. EC: endothelial cells. Grn: glucoraphanin. GSH: glutathione. HMEC: human microvascular endothelial cells. HUVEC: human umbilical vein endothelial cells. IL: interleukin. i.p.: intraperitoneal injection. I/R: ischemia-reperfusion. i.v.: intravenous injection. RIN cells: rat pancreatic *β*-cell line RINm5F. SHRsp: spontaneously hypertensive stroke-prone rats. SMC: smooth muscle cell. STZ: streptozocin. TNF-*α*: tumor necrosis factor-*α*. VSMC: vascular smooth muscle cell. T2DM: type 2 diabetes mellitus. VCAM: vascular cell adhesion molecule. HAEC: human aortic endothelial cells. DCM: diabetic cardiomyopathy.
